# Bone-targeted lipoplex-loaded three-dimensional bioprinting bilayer scaffold enhanced bone regeneration

**DOI:** 10.1093/rb/rbae055

**Published:** 2024-06-03

**Authors:** Woo-Jin Kim, Jeong-Hyun Ryu, Ji Won Kim, Ki-Tae Kim, Hye-Rim Shin, Heein Yoon, Hyun-Mo Ryoo, Young-Dan Cho

**Affiliations:** Department of Molecular Genetics, School of Dentistry and Dental Research Institute, Seoul National University, Seoul, 03080, Republic of Korea; Department of Molecular Genetics, School of Dentistry and Dental Research Institute, Seoul National University, Seoul, 03080, Republic of Korea; Department of Molecular Genetics, School of Dentistry and Dental Research Institute, Seoul National University, Seoul, 03080, Republic of Korea; Department of Molecular Genetics, School of Dentistry and Dental Research Institute, Seoul National University, Seoul, 03080, Republic of Korea; Department of Molecular Genetics, School of Dentistry and Dental Research Institute, Seoul National University, Seoul, 03080, Republic of Korea; Department of Molecular Genetics, School of Dentistry and Dental Research Institute, Seoul National University, Seoul, 03080, Republic of Korea; Department of Molecular Genetics, School of Dentistry and Dental Research Institute, Seoul National University, Seoul, 03080, Republic of Korea; Department of Periodontology, School of Dentistry and Dental Research Institute, Seoul National University and Seoul National University Dental Hospital, Seoul, 03080, Republic of Korea

**Keywords:** 3D bioprinting, bone-targeting lipoplex, BMP2, bone regeneration

## Abstract

Clinical bone-morphogenetic protein 2 (BMP2) treatment for bone regeneration, often resulting in complications like soft tissue inflammation and ectopic ossification due to high dosages and non-specific delivery systems, necessitates research into improved biomaterials for better BMP2 stability and retention. To tackle this challenge, we introduced a groundbreaking bone-targeted, lipoplex-loaded, three-dimensional bioprinted bilayer scaffold, termed the polycaprolactone-bioink-nanoparticle (PBN) scaffold, aimed at boosting bone regeneration. We encapsulated BMP2 within the fibroin nanoparticle based lipoplex (Fibroplex) and functionalized it with DSS_6_ for bone tissue-specific targeting. 3D printing technology enables customized, porous PCL scaffolds for bone healing and soft tissue growth, with a two-step bioprinting process creating a cellular lattice structure and a bioink grid using gelatin-alginate hydrogel and DSS_6_-Fibroplex, shown to support effective nutrient exchange and cell growth at specific pore sizes. The PBN scaffold is predicted through *in silico* analysis to exhibit biased BMP2 release between bone and soft tissue, a finding validated by *in vitro* osteogenic differentiation assays. The PBN scaffold was evaluated for critical calvarial defects, focusing on sustained BMP2 delivery, prevention of soft tissue cell infiltration and controlled fiber membrane pore size *in vivo*. The PBN scaffold demonstrated a more than eight times longer BMP2 release time than that of the collagen sponge, promoting osteogenic differentiation and bone regeneration in a calvarial defect animal. Our findings suggest that the PBN scaffold enhanced the local concentration of BMP2 in bone defects through sustained release and improved the spatial arrangement of bone formation, thereby reducing the risk of heterotopic ossification.

## Graphical abstract

**Figure:**
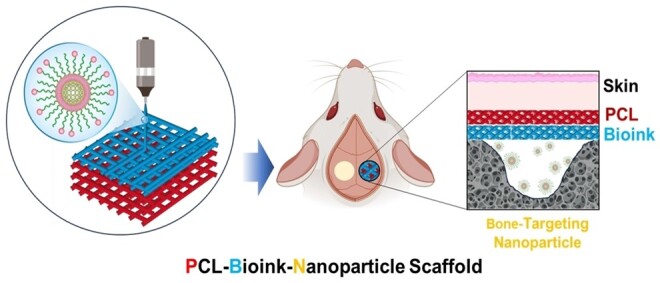

## Introduction

The treatment of bone morphogenetic proteins (BMP) for addressing large-scale bone defects is protein established medical engineering therapeutics recently employed extensively in clinical settings. External BMP treatment stimulates the cellular pathways that enhance bone formation in both non-human and human subjects [1]. Following the introduction of BMP2 into the medical market, the application of clinically approved products containing BMP2 has surged, comprising 20–25% of all spinal fusion procedures [2]. Subsequently, both on- and off-label utilization increased, with the estimated proportion of off-label usage in major surgeries reaching ∼85% [3].

While clinical BMP2 delivery is effective in stimulating bone growth, its use, especially in off-label spinal procedures, has led to issues like soft tissue swelling and unintended bone formation in ∼28% of patients [4]. It is thought that these complications occur as a result of the use of supraphysiological doses of BMP2 required for effective human treatment, necessary to counteract the protein’s brief half-life of around ∼6–7 minutes and its quick elimination from the body [5] and non-bone-specific drug delivery system [6]. The standard dosages of clinical BMP2 vary from 0.05–0.6 mg/kg (milligrams per kilogram) of body mass, with reports of off-label use reaching up to 1.0 milligram of BMP2 per kilogram [7]. Considering the rapid removal of BMP2 from the body, clinical BMP2 delivery usually involves adsorbing the protein onto a collagen sponge-based scaffold and localizing it at the site of the bone defect [8]. However, a wide range of research has shown that collagen sponges do not possess a specific affinity for BMP2, have no beneficial effect on bioactivity, are unable to retain the protein effectively within the matrix (<10%), and could potentially hinder fracture healing if applied without BMP2 [9, 10]. As a result, substantial research endeavors are focused on creating better biomaterials designed to enhance the stability and *in vivo* retention of BMP2.

Hydrogel bioinks have been widely studied for biomaterial delivery owing to broad applicability in three-dimensional (3D) bioprinting using printers, injectables and modifiable chemicals to control degradation and release profile and permeability to cells and macromolecules [11, 12]. Bioinks primarily utilize substances such as fibrin, collagen, polyethylene glycol, gelatin, hyaluronic acid and alginate. They rely on mechanisms of attachment that include covalent binding strategies and nonspecific electrostatic interactions [13–16]. Nevertheless, polymerization reactions triggered by ultraviolet (UV) light or heat frequently require the chemical alteration of the protein, potentially leading to reduced bioactivity [17]. Moreover, the physicochemical properties of the hydrogels following the induction of suitable polymerization reactions impact printability, manipulation and other factors.

The high loading and long-term release of protein drugs are crucial characteristics of protein drug delivery systems. However, the size and charge properties of proteins as macromolecules make it difficult to incorporate them at high capacities into conventional polymer-based nanoparticles, hindering the ability to control long-term protein drug release while maintaining high loading. We have developed a silk fibroin protein nanoparticle system coated with a cationic liposome surface called Fibroplex, which enables high-density growth factor loading and long-term stable release [18]. This Fibroplex demonstrated an improved physicochemical profile, displaying a release profile with long-term stability of over 30 days while incorporating high protein capacities [19]. The substantial BMP2 loading capacity and extended release profile of Fibroplex render it an optimal delivery mechanism for the administration of the supraphysiological doses of BMP2 needed in clinical contexts. For bone-tissue targeting, two types of molecules, bisphosphonates and oligopeptides, including eight repetitive sequences of aspartate (Asp8) and six repeated amino acids of the sequence aspartate-serine-serine (DSS_6_), have been studied [20]. Bisphosphonate is usually distributed at both bone defects and resorption region; however, Asp8 preferentially binds to the bone resorption site, and DSS_6_ has high specificity for bone formation surface [21].

In this study, we encapsulated BMP2 at high capacity within a Fibroplex and functionalized it with DSS_6_ for bone tissue-specific targeting. We dispersed the DSS_6_-functionalized Fibroplex in a hydrogel bioink composed of gelatin and alginate and bioprinted it as an osteoconductive biomaterial surrounded by a polycaprolactone (PCL) nanofiber mesh (PCL-Bioink-Nanoparticle Scaffold; PBN scaffold). To evaluate the efficacy of the PBN scaffold, we examined the effectiveness of a composite biomaterial delivery system for prolonged BMP2 delivery to critical calvarial defects. We focused on preventing the growth and infiltration of soft tissue cells into the bone defect area by controlling the pore size of the fiber membrane *in vivo* while maintaining physiological BMP2 concentrations at the defect site during the bone formation period through the sequential degradation of the hydrogel bioink and Fibroplex. Using this approach, we hypothesized that we could localize the BMP2 release within binding site of PBN scaffold, enhance the local concentration of BMP2 in the bone-defect area to physiological levels and improve the spatial distribution of bone regeneration.

## Materials and methods

### Materials

Unless specified otherwise, Sigma-Aldrich supplied chemicals used as is. This includes Polyvinyl alcohol (PVA) with a molecular weight between 30 000 and 70 000, alginate (low viscosity: 3–15 cP, 1.0% in deionized distilled water (DDW)), collagen-gelatin (gel strength: 3–400 g Bloom, type A from porcine skin), calcium chloride (CaCl_2_, anhydrous, granular, ≤10 mm, ≥95.0% purity) among others, all purchased from Sigma-Aldrich. The study also utilized proteins, which were produced in Escherichia coli BL21 cells that had been genetically modified and then isolated through nickel-resin affinity chromatography, a process also facilitated by Sigma-Aldrich. Additional materials like DOTAP, DOPE was sourced from Avanti Polar Lipids in Birmingham, AL, USA. For all experiments, DDW from a Milli-Q system provided by Millipore, was utilized.

### Fabrication of fibroin nanoparticles

Silk fibroin nanoparticles were produced using a revised method based on the procedure outlined by Kim and colleagues [18]. Bombyx mori cocoons were boiled, rinsed and dried before being dissolved in LiBr solution. The solution was dialyzed, centrifuged and adjusted to 8% (w/v) concentration. Silk fibroin (mean MW 370 kda) and PVA solutions (5 wt%) were blended in a 1/4 weight ratio, sonicated and transferred to Petri dishes for drying. The dried silk/PVA films were dissolved in DDW, and the pellets were cleaned, resuspended in PBS and sonicated to disperse the particles.

### BMP2 encapsulated in fibroin nanoparticles

Enhanced green fluorescence protein (GFP) and BMP2, was applied as loading drug to reveal loading in fibroin particles. Stock solutions (500 µM) were prepared in PBS and stored at −20°C. The model drugs were combined with the fibroin stock solution at various ratio and mixed with the PVA stock solution according to the fibroin particle preparation method. Model drug loading in the nanoparticles was determined by analyzing the supernatants of centrifugation stages using enzyme-linked immunosorbent assay (ELISA). Loading protein content in the nanoparticles determined by calculating the difference between the input protein concentration and the level of present in supernatant. The final pellets were resuspended in PBS for further analysis. For the determination of the loading efficiency of BMP2, the remaining BMP2 in the supernatant was measured. Amount of BMP2 were analyzed using an ELISA kit, following the guidelines provided by the manufacturer.

Loading efficiency of BMP2 = (actual loaded amount of BMP2)- (BMP2 in the supernatant)/(actual loaded amount of BMP2) × 100 (%).

### Functionalization of DOPE lipid with DSS_6_

To functionalize the DOPE lipids with a peptide using PEG2000, as a linker, the following process was performed. First, DOPE lipids were obtained in chloroform solution (Cat. 850725P, AvantiLipid, Germany). Next, a heterobifunctional PEG linker such as DSPE-PEG2000-N-hydroxysuccinimide (NHS) (Cat. 880138P, AvantiLipid, Genrmany) was added to the head group of DOPE; the NHS ester group in the PEG linker reacted with the amine group of the DOPE lipids. Chloroform was removed from the lipid mixture by either using a rotary evaporator or by gently passing nitrogen gas over the solution, leading to the creation of a thin film of lipid on the container's walls. The dried lipid film was rehydrated by adding PBS or another suitable buffer and vortexed to create a liposome suspension. To achieve consistent liposome size, the lipid-nanoparticle suspension was passed through a filter (polycarbonate) membrane with a defined 100 nm porosity. Additionally, a solution containing the target peptide, which has a primary amine group capable of reacting with the NHS ester group on the PEG2000 linker, was prepared. The peptide solution was mixed with the suspension of PEGylated liposomes and allowed to incubate for several hours or overnight at room temperature. This step facilitates the conjugation of the peptide to the PEG2000 linker. Finally, any unreacted peptide was removed by dialysis, size-exclusion chromatography or ultracentrifugation and purified peptide-functionalized DOPE liposomes were collected for further experiments.

### Preparation and distribution of gelatin-alginate hydrogel bioink

The preparation of the gelatin-alginate hydrogels was conducted using a blend of previous report [10]. Briefly, the gelatin solution (Gel; 20%, w/v) and sodium alginate solution (Alg; 8%, w/v) were autoclaved before the mixing process which was strictly sterilized. The Gel-Alg solution was mixed to 6 ml 20% (w/v) Gel solution and 3 ml 8% (w/v) Alg solution. To fabricate the Gel-Alg composite hydrogel, the Gel-Alg solution was placed into the cylindrical mold (diameter 4 mm; height 0.5 mm) at 0°C. Gel-Alg composite hydrogel which was separated from the cylindrical mold was immediately immersed in 2 ml sterilized calcium chloride 10% (w/v) solution ∼10 min at 0°C. To confirm encapsulation efficiency of nanoparticles within the Gel-Alg composite hydrogel, 10 ml of Gel-Alg composite solution with 1 mg of nanoparticles (1:10% w/v) was gently mixed. The Gel-Alg composite hydrogel containing nanoparticles was incubated for 1 day under 4°C. To detect the distribution of encapsulated nanoparticles, nanoparticles were loading with FITC then observed by a fluorescence microscope (MetaFluor, Molecular Devices).

### Preparation of 3D printed implants

PCL (polycaprolactone; molecular weight of 45 000) was utilized for the fabrication of 3D printable structure in this study (Cat.704105, Sigma Aldrich, US). PCL Scaffolds were produced using a custom-built pneumatic fused deposition system, which was installed on a device equipped with a 100 μm in diameter nozzle (DR INVIVO 4D2 Bioprinter, ROKIT Healthcare, Korea). Briefly, Three-dimensional image files were designed on cylindrical shapes (4 mm diameter and 1 mm height) using NewCreatorK (ROKIT Healthcare, Seoul, Republic of Korea) software. The stereolithography files were converted into G-code instructions suitable for the 3D printer. Following the upload of the G-code file to the 3D printer, specimens were printed to fabricate the scaffold. The PCL scaffold was constructed with specific parameters: Print temperature of 83°C, a bed temperature of 24°C, a fill density of 10–30%.

### *In vitro* release profile study

Fibroplexes containing model drugs were dispersed in PBS with gentle shaking. After that, the samples were centrifuged at 18 000 rpm for 15 minutes using an Eppendorf microcentrifuge (Model 5417R). The supernatants were meticulously transferred to fresh tubes, and the pellets were re-dissolved in 1 ml of PBS for ongoing release experiments. The optical density of the collected supernatants was measured at 555 nm to determine the amount of drug not absorbed, utilizing a standard curve for the calculations. Cumulative release data were determined by relating the measurements to the initial loading amount in the fibroplexes. For each proteins, a minimum of three duplicates were analyzed to ensure statistical reliability.

### *In vitro* hydroxyapatite and calcium salt binding assay

The binding assay for hydroxyapatite and calcium salts was adapted from prior research. Hydroxyapatite crystals (HA, molecular weight (MW) 502 g/mol), calcium carbonate (CC, MW 100 g/mol), calcium pyrophosphate (CPP, MW 254 g/mol) and calcium phosphate (CP, MW 310 g/mol) salts were incubated with DSS_6_-Fibroplex and Fibroplex-alone (as a negative control) at a concentration of 5 μg/mL in PBS. The residual nanoparticles were removed five times with 0.05% Phosphate Buffered Saline with Tween 20 (PBST) through centrifuged under 18 000 rpm, then transferred to a 96-well plate for evaluation using a fluorimeter.

### Morphometric analysis of scaffold

The mean pore size for PBN scaffold was determined using a modified method based on previous study [22]. Briefly, the process began with the selection of a region of interest, which encompassed a particular strand. The boundaries of that strand were delineated using ImageJ software. The software then measured perpendicular lines extending from one boundary to the opposite side. To ascertain pore size values, the gap between two adjacent strands was selected as the region of interest (ROI). This procedure was replicated until the software calculated the average size of distance between strands across various SEM results to obtain the mean pore size.

### Development of BMP2 dynamics prediction

A prediction algorithm for *in vivo* BMP2 release from defect sites was created using COMSOL Software (version 5.2a). The software depicted the 3D bone defect and its adjacent tissue as various overlapping rectangles, axisymmetric space. Therefore, the model's subdomains were designated as (i) calvarial defects and (ii) the defect-surrounding soft tissue. To simulate interactions between BMP2 and the bioink, the reaction engineering physics module was employed, delivery of dilute species physics module was utilized for represent the release profile of BMP2 through the bioink and into the adjacent area. The formulas describing BMP2 diffusion and its interactions with the bioink are grounded in Fick's diffusion laws, behalf straightforward binding kinetics. The effective diffusion coefficient of BMP2 through the bioink (*D*_bioink_) was concluded based on *in silico* BMP2 release profile via an 8% (w/v) bioink in capillary tubes. The effective diffusion coefficient of released BMP2 through the bone defect (*D*_Bone tissue_) and the surrounding soft tissue (*D*_soft tissue_) was approximated using fluorescent BMP2 tracking at the bone defect site. Rate constants were initially chosen based on BMP2 binding to collagen sponge (*K_d_* = 20 nM, *k*_on_ = 5 × 1 0^−4^ 1/nM⋅s and *k*_off_ = 0.01 1/s) (1) and then, refined for soft tissue by employing a conventional model for *in silico* BMP2 diffuse modeling, as described above, revealed a greater dissociation rate constant than that of soft tissue was necessary to model the experimental results accurately (*K_d_* = 50 nM, *k*_on_ = 5 × 1 0^−4^ 1/nM⋅s and *k*_off_ = 0.025 1/s). Sensitivity analysis was conducted via altering various parameters through factors of 2.5, 5 or 10: (i) the *K_d_* characterizing BMP2, (ii) the diffusion coefficient for both soft and bone tissues and (iii) the diffusion coefficient across each tissue's properties [23].

### *In vitro* cytotoxicity assay

The *in vitro* cytotoxicity of scaffold was evaluated using the MTT assay. MC3T3-E1 cells were cultured at 8000 cells per well in a 96-well plate, one day before the introduction of Fibroplexes. These cells underwent treatment with various concentrations of Fibroplexes for a duration of 12 hours. After this treatment period, the Fibroplexes were removed and the cells were subsequently cultured in fresh medium for varying lengths of time. Following incubation, 20 μl of MTT solution was applied to each well and allowed to incubate for adjust colorimetric. The medium was then aspirated away, and 200 μl of dimethyl sulfoxide (DMSO) was added to dissolve the formazan crystals. The plate was placed on a shaker at 150 rpm to ensure thorough mixing. The absorbance of samples was analyzed at 560 nm to assess cell viability, with untreated cells serving as the control for 100% cell proliferation.

### *In vitro* osteogenic differentiation

MC3T3-E1 cells were utilized to assess their osteogenic potential, with BMP2 released from the PBN scaffolds applied to the cells for durations ranging from 1 to 7 days. RNA extraction was performed using QIAzol lysis reagent (Qiagen, Valencia, CA, USA). For reverse transcription, the PrimeScript RT reagent kit was acquired from TAKARA (Shiga, Japan). The evaluation of murine ALP, Runx2, Col1a1 and GAPDH expression was analyzed via quantitative real-time polymerase chain reaction (RT-qPCR), employing primers previously established in the literature. Each sample was analyzed in duplicate to ensure accuracy, and the relative mRNA levels were normalized to the levels of GAPDH to ascertain gene expression variations accurately.

ALP staining was performed as previously described, and the released BMP2 was used to treat MC3T3-E1 cells seeded in 12-well culture plates for 4 days. Cells were rinsed with PBS and incubated with ALP solution according to the manufacturer’s guide.

### *In vivo* calvarial defect model

In this study, 12 C57BL/6 mice (8 weeks of age) were utilized. The mice were anesthetized using a mixture of Zoletil (0.4 ml/kg) and Rumpun (10 mg/kg). After shaving and applying an antiseptic dressing, the epicranium was cut longitudinally. A 4 mm diameter trephine bur was used to create critical-sized calvarial defects without dural perforation under constant irrigation with physiological saline. In addition, a 4 mm diameter collagen sponge (4 mm × 0.5 mm height) soaked with BMP2 (0.5 μg) or PBN scaffold (0.5 μg of BMP2) was used as previously described in the *in vivo* study (*N* = 4 per group). The excised periosteum was meticulously removed, and a collagen sponge was then placed into the defect site. Following this, both the superficial epithelial layer and the skin were stitched closed. Healing capacity of bone defects was measured at 2-, 4- and 8-weeks post-implantation. MicroCT analysis was performed as previously described [24]. Quantitative analysis was performed using a CT analyzer (Bruker) and TriBON™ software (RATOC System Engineering Co.). All animal experiments performed in this study were approved by the Animal Care and Use Committee of Seoul National University under animal protocol number SNU-210202-5-3.

### Statistics

All quantitative data are presented as means ± SD. Each experiment was performed at least three times. Results from representative individual experiments are shown in the figures. Statistical analyses were performed using a one-way analysis of variance followed by Bonferroni's test or Student's *t*-test using Prism software (version 5.0; GraphPad Software). A *P* values < 0.05 was considered statistically significant.

## Results

### Fabrication of bone-targeting lipoplex loaded 3D printed scaffolds

**Figure 1.**
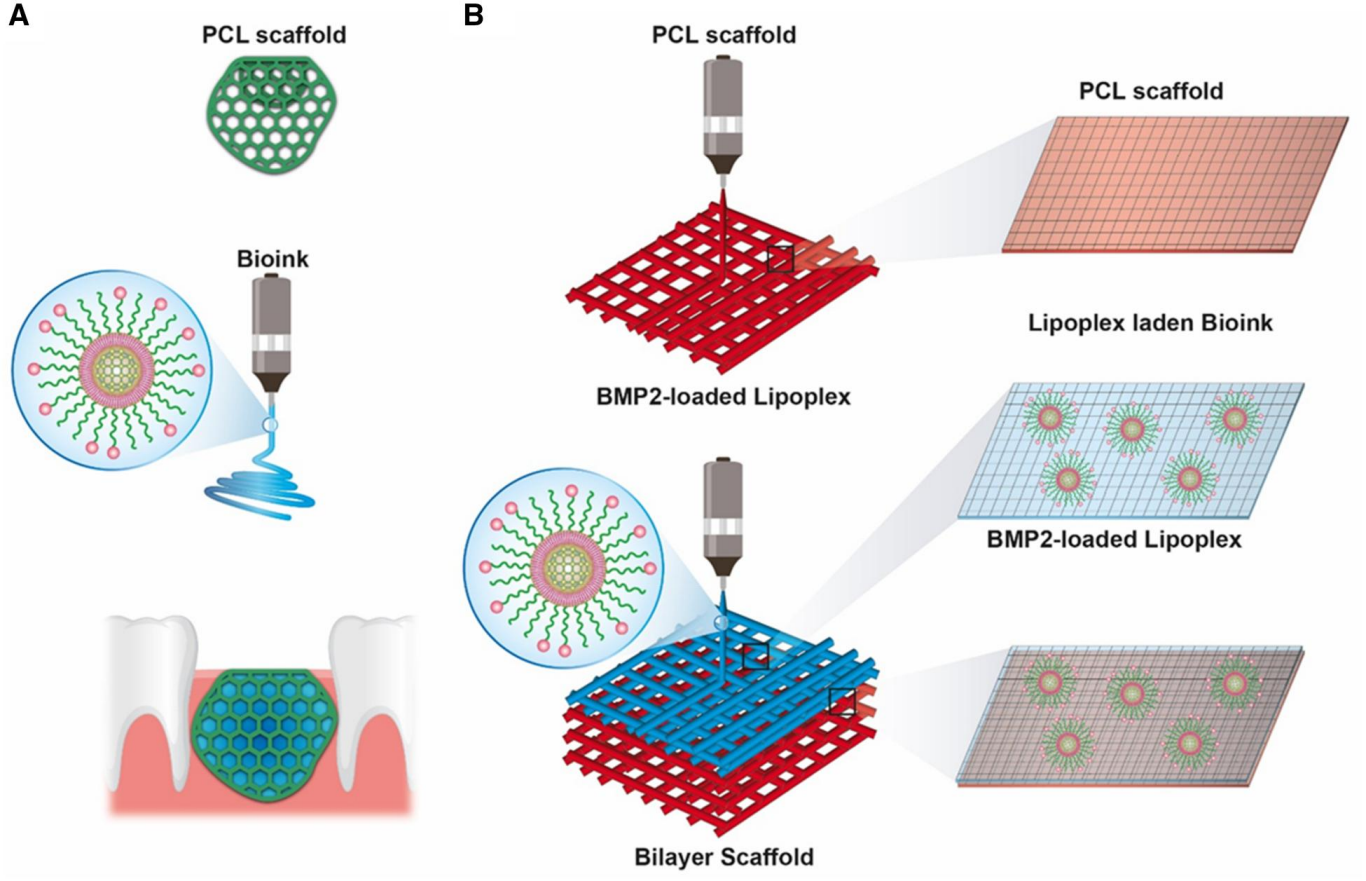
Schematic description and fabrication of bone-targeting lipoplex-loaded 3D-printed scaffolds. (**A**) Schematic of the PBN scaffold. The PCL scaffold faces the soft tissue side, and the bioink containing the lipoplex is positioned toward the bone defect area. (**B**) Fabrication process and structure of the PBN scaffold. 3D, three-dimensional; PBN, polycaprolactone-bioink-nanoparticle; PCL, polycaprolactone.

We used a PCL scaffold that directly contacts the soft tissue and provides mechanical strength to the hydrogel bioink. Bone-targeting functionalized lipoplexes were introduced into the hydrogel bioink, which were then attached to one side of the PCL scaffold to complete the PBN scaffold. The PBN scaffold offers an optimal pore size and structure conducive to the proliferation of soft tissue, simultaneously serving as a barrier against the soft tissue ingrowth into the bony defect. On the other hand, the lipoplexes with bone tissue-specific targeting ability are stably released from the bioink toward the bone injury site, and the BMP2 encapsulated in the lipoplexes maintains a physiological concentration over an extended period at the bone surface (Figure 1A).

We created a PCL scaffold utilizing a pneumatic fused deposition system that was installed on an additive manufacturing apparatus, equipped with a nozzle measuring 100 μm in diameter. The hydrogel bioink containing BMP2-loaded bone-targeting lipoplex was then printed on the PCL scaffold using the same pattern as that of the PCL scaffold (Figure 1B). The resulting PBN scaffold has dual functionality: one side with the PCL scaffold suitable for soft tissue layer proliferation with an appropriate pore size and the other side functionalized for bone-targeting drug delivery.

### Fabrication and characterization of bone-targeting nanoparticle-liposome complex

**Figure 2.**
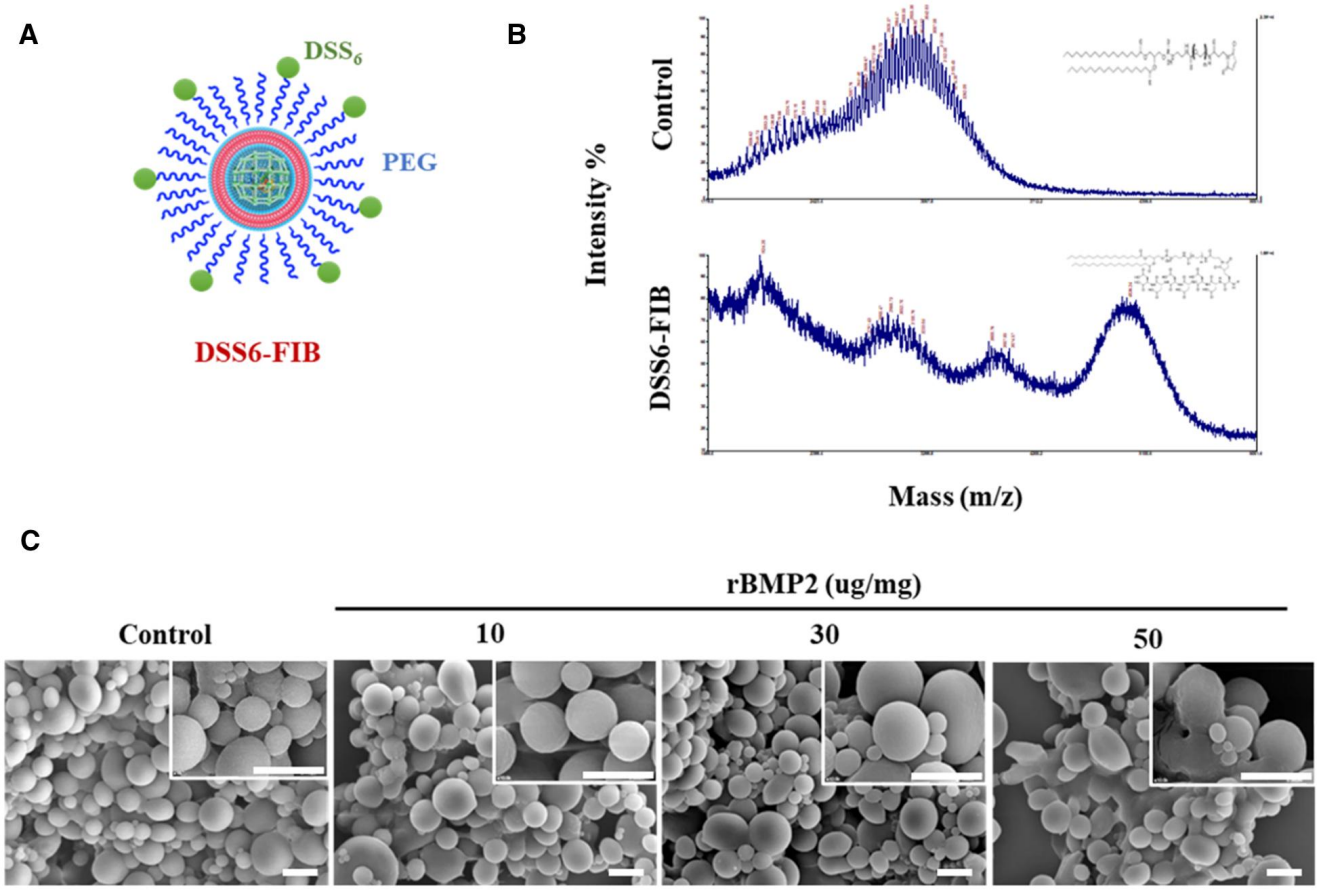
Characterization of bone-targeting functionalized fibroin particle encapsulated cationic lipid complex. (**A**) Schematic representation of the surface functionalization structure of the Fibroplex. (**B**) MALDI-TOF analysis results of DSS_6_ for Fibroplex functionalization. (**C**) Concentration optimization of BMP2 introduced into the Fibroplex. Each scanning electron microscopic image is magnified ×5000 (enlarged right corners = ×10 000). BMP2, bone morphogenetic protein 2; MALDI-TOF, matrix-assisted laser desorption/ionization-time of flight.

Fibroin nanoparticles and cationic lipid complexes (Fibroplex) are delivery platforms capable of high-capacity loading and sustained release of protein drugs. Cationic liposomes are composed of a mixture of the 1,2-dioleoyl-3-trimethylammonium-propane (DOTAP) and dioleoylphosphatidylethanolamine (DOPE). We functionalized lipid nanoparticles with DSS_6_ (a bone-specific docking peptide) using PEG2000 as a linker on the surface of DOPE to provide bone-specific targeting functionality (DSS_6_-Fibroplex). As a result, DSS_6_-Fibroplex can enhance its ability to deliver specifically to bone tissue (Figure 2A).

MALDI-TOF analysis was performed for the peptide sequencing on the surface of the fabricated DSS_6_-Fibroplex showed the presence of the peptide in the DSS_6_-Fibroplex, whereas no peptide was observed in the control (Figure 2B). Average size of DSS_6_-Fibroplex was 551 nm, with a PI of 0.279 (Supplementary Figure 1). The introduction of recombinant BMP2 (BMP2) protein into the DSS_6_-Fibroplex did not significantly affect the carrier size and stability. The amount of BMP2 introduced into the fibroplex increased with concentration. Upon increasing the amount of BMP2, it was observed that when 50 μg of BMP2 was introduced per mg of DSS_6_-Fibroplex, an increase in PI and particle instability was observed (Figure 2C and Supplementary Figure 2). The results from measuring size and surface potential using a zetasizer also corroborated as the incorporation of BMP2 was increased from 5 to 40 μg/mg. (Supplementary Figure 3). Therefore, we determined the BMP2 content in DSS_6_-Fibroplex to be 30 μg per mg and proceeded with subsequent experiments. In the *in vitro* calcium salt binding assay results, fibroplex modified with DSS_6_ exhibited ∼18 times higher binding affinity for hydroxyapatite compared to unmodified fibroplex and also showed significantly higher binding affinity for calcium phosphate. However, there was no notable difference in binding affinity for collagen or calcium carbonate (Supplementary Figure 4).

### Characterization of PCL-bioink-nanopaticle scaffold (PBN scaffold)

**Figure 3.**
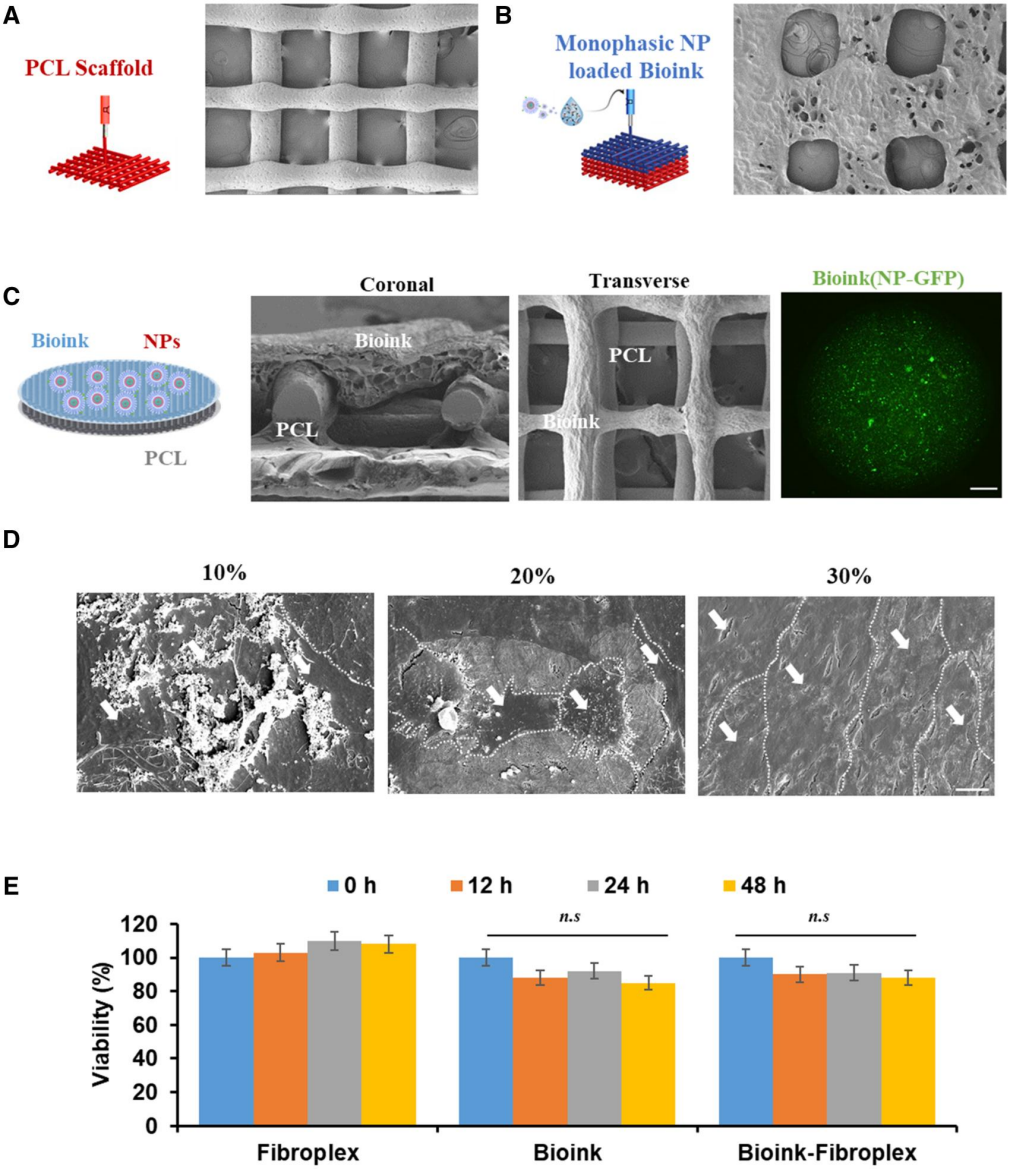
Structure of PCL-bioink-nanoparticle scaffold and its biocompatibility. (**A**) Scanning electron microscope (SEM) image of PCL scaffold. Scale = 100 μm. (**B**) SEM image of bioink. Scale = 200 μm. (**C**) SEM image of PBN scaffold (left) and confocal microscope image of GFP-loaded Fibroplex dispersed in bioink (right). (**D**) SEM images for observing the attachment and growth of NH3T3 cells on PBN scaffolds with various filling densities (10–30%). White arrows indicate individual cells. (**E**) Cytotoxicity of each component of the PBN scaffold. PCL, polycaprolactone; PBN, polycaprolactone-bioink-nanoparticle; GFP, green fluorescence protein.

The PCL scaffold was crafted into a cellular lattice design resembling the bone matrix, featuring a three-dimensional porous spatial configuration. It was constructed by layering in a 0/90° orientation, utilizing continuous contour filaments to mimic the natural structure of bone (Figure 3A). The PCL scaffold was first cooled to room temperature after printing two layers of thermoplastic PCL. Then, the same pattern of gelatin-alginate hydrogel was printed on top, followed by cross-linking with calcium chloride (CaCl_2_). In this process, the gelatin-alginate hydrogel was developed as a biocompatible bioink that could maintain the stability of DSS_6_-Fibroplex to a maximum and was found to have a viscosity and elastic modulus that allowed stable printing on the PCL scaffold (Supplementary Figure 2). BMP2-loaded DSS_6_-Fibroplex was dispersed in the gelatin-alginate hydrogel at 5–20% (v/v). X-ray diffraction analysis showed that DSS_6_-Fibroplex was detected in all groups. However, the elastic modulus tended to increase as the proportion of DSS_6_-Fibroplex increased, showing a sharp increase in the loss modulus at 10% and above (Supplementary Figure 5). The DSS6-Fibroplex-loaded bioink, with a 10% dispersion ratio determined based on the appropriate physical properties, successfully formed a mesh structure in the independent printing results (Figure 3B and Supplementary Figure 6).

The scanning electron microscope (SEM) image of the PBN scaffold, which has a mixed layer of scaffold and bioink, shows microstructural features of PBN scaffold (Figure 3C). The two-step bioprinting uniformly formed a bioink grid, forming an independent layer on the PCL; in particular, the introduced green fluorescence protein-loaded DSS_6_-Fibroplex showed uniformly dispersed fluorescence in the cross-section and top-view of the bioink single layer (Supplementary Figure 7).

PCL scaffolds fabricated with filling densities of 10%, 20% and 30% demonstrated interconnected pores with respective diameters of 46 ± 4 μm, 174 ± 51 μm and 238 ± 42 μm, showcasing the scaffold's ability to support tissue growth through its porous structure. When fibroblasts were cultured on the PCL phase of each PBN scaffold, the cells did not grow at 10% filling density but formed an appropriate cell layer and grew at 20% and 30% (Figure 3D). This aligns with findings from earlier research, which indicate that scaffold pores ranging in diameter from 300 to 1200 μm are optimal for promoting cell viability. These results are consistent with the measurements of the overall or individual component cytotoxicity of the PBN scaffold (Figure 3E).

### PBN scaffolds are predicted to increase BMP2 release in bone defect *in silico*

The expectation was that various interfaces of the PBN scaffold would modify the BMP2 release pattern; hence, prediction strategy was devised using COMSOL Multiphysics software to forecast BMP2 diffuse at the defect site.

**Figure 4.**
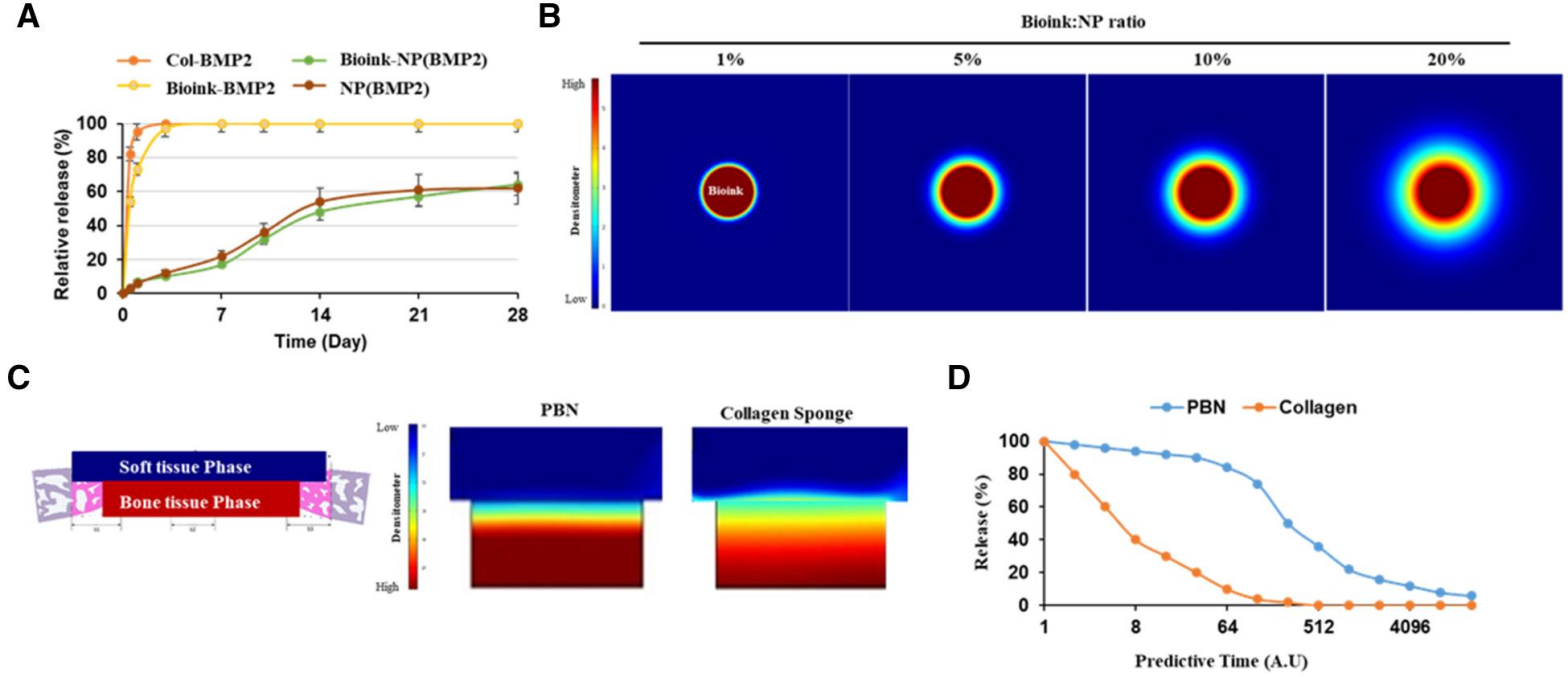
*In silico* assessment of BMP2 release modeling. (**A**) ELISA assay measuring BMP2 release rate for each component of the PBN scaffold. (**B**) Predicted release patterns of BMP2 dispersed in the bioink. Bar = densitometer reflecting the predicted concentration. (**C**) Predicted BMP2 release pattern, assuming a high-density boundary (soft tissue phase) and a low-density boundary (bone defect phase). (**D**) Tissue release pattern curve predicted through modeling. BMP2, bone morphogenetic protein 2; PBN, polycaprolactone-bioink-nanoparticle; ELISA, enzyme-linked immunosorbent assay.

A sensitivity analysis was carried out on a number of critical parameters within the COMSOL model, specifically, the dissociation constant (*K*_d_) of BMP2 from the PBN scaffold, the BMP2 diffusion coefficient in soft tissue (*D*_Soft Tissue_) and the BMP2 diffusion coefficient within the bone tissue defect (*D*_Bone Tissue_), utilizing the diffusion coefficient measurement results under various conditions of the PBN scaffold. The values for *K*_d_, *D*_Soft Tissue_ and *D*_Bone Tissue_ were adjusted from ±1.8 to 12 times their baseline figures. BMP2 release was 18–21% at 7 days, 48–54% at 14 days, 57–61% at 21 days and 62–64% at 28 days, depending on the scaffold conditions, whereas the control group showed a 100% release rate under the same conditions (Figure 4A). Therefore, it was anticipated that the overall BMP2 release would be chiefly influenced by the variance in BMP2 diffusion rates, which are dependent on the detachment dynamics of BMP2, the makeup of the scaffold and the density of the tissue surrounding it. Based on the COMSOL model, we confirmed that the BMP2 diffusion coefficient had a low impact owing to the bioink. BMP2 diffusion in the bioink was predicted to occur in a concentration-dependent manner (Figure 4B).

Visual representation of the predicted BMP2 release amounts from the PBN scaffolds and collagen sponges on both tissue surfaces differed depending on the tissue-specific values (Figure 4C). When predicted based on the 28-day release amount, the PBN scaffold simulation at the soft tissue boundary only showed differential BMP2 release towards the bone-defect side, and the BMP2 release amount was predicted to be higher. In contrast, the control group collagen sponge showed weak release towards the soft tissue surface (Figure 4C). In the predicted results for the BMP2 release profile, the PBN scaffold simulated a release time that was eight times longer than that of the collagen sponge based on a lower K_d_ with BMP2 (Figure 4D).

### Sustained released BMP2 in PBN scaffold promotes osteogenesis differentiation

**Figure 5.**
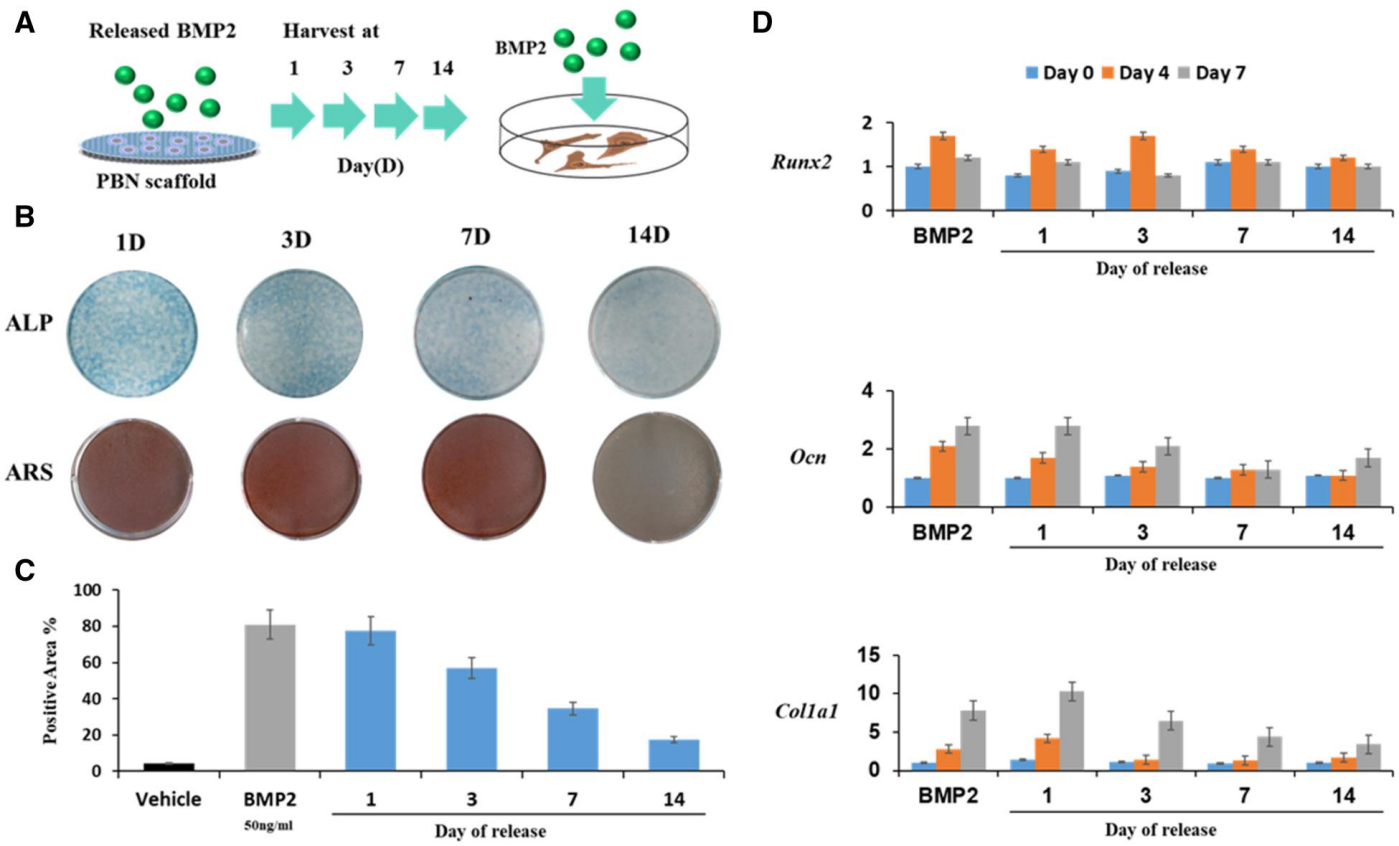
Osteogenic inductive efficacy of BMP2 from the PBN scaffold. (**A**) Strategy for the acquisition of BMP2 released from the PBN scaffold over time. (**B**) ALP staining and Alizarin Red S (ARS) staining images of MC3T3-E1 cells treated with BMP2 collected at each time point after 4 days and 21 days, respectively. (**C**) ALP assay results of MC3T3-E1 cells treated with BMP2 collected at each time point after 4 days. (**D**) qPCR results of osteogenic markers in MC3T3-E1 cells treated with BMP2 collected at each time point after 4 and 7 days. *P* < 0.05, all biological replicates are *N* = 4. BMP2, bone morphogenetic protein 2; PBN, polycaprolactone-bioink-nanoparticle; ALP, alkaline phosphatase; qPCR, quantitative polymerase chain reaction.

We investigated whether the BMP2 encapsulated in the PBN scaffold exhibited a simulated sustained-release profile. One milligram of the BMP2-introduced PBN scaffold was soaked in 1 ml of culture medium and allowed to release BMP2 for 1, 3, 7 and 14 days. Diffused cargos were then treated on cultured MC3T3E1 preosteoblast cells to verify its ability to induce bone differentiation (Figure 5A).

Alkaline phosphatase (ALP) staining was performed to analyze whether the released BMP2 in the medium collected at each time point contained an effective concentration capable of inducing bone differentiation in preosteoblasts. ALP activity was confirmed in all groups, and effective results were observed even 14 days after release. Additionally, Alizarin Red S staining (ARS) performed after induction of late-stage of differentiation also showed a similar pattern (Figure 5B). Quantitative analysis showed that ALP activity was the highest on day 1, displaying a similar level to that of positive control with BMP2 treatment (Figure 5C). In Figure 4A, the cumulative BMP2 release profile was investigated to determine if BMP2 release sustains protein activity over time. The accumulated BMP2 was collected at various time points and assessed through ALP staining, revealing increased ALP activity corresponding to the amount of BMP2 released (Supplementary Figure 8).

Release profile of BMP2 from PBN scaffold stimulates the activation of genes marking bone differentiation, specifically *Runx2*, *Ocn* and *Col1a1*. Pre-osteoblast cells were treated with BMP2 collected at each release time point, and bone differentiation was induced for up to 7 days. Subsequently, measurement of the mRNA levels of Runx2, Ocn and Col1a1 on days 4 and 7 after the induction of bone differentiation revealed a significant increase (Figure 5D).

### PBN scaffold promotes bone regeneration in calvarial defect model

**Figure 6.**
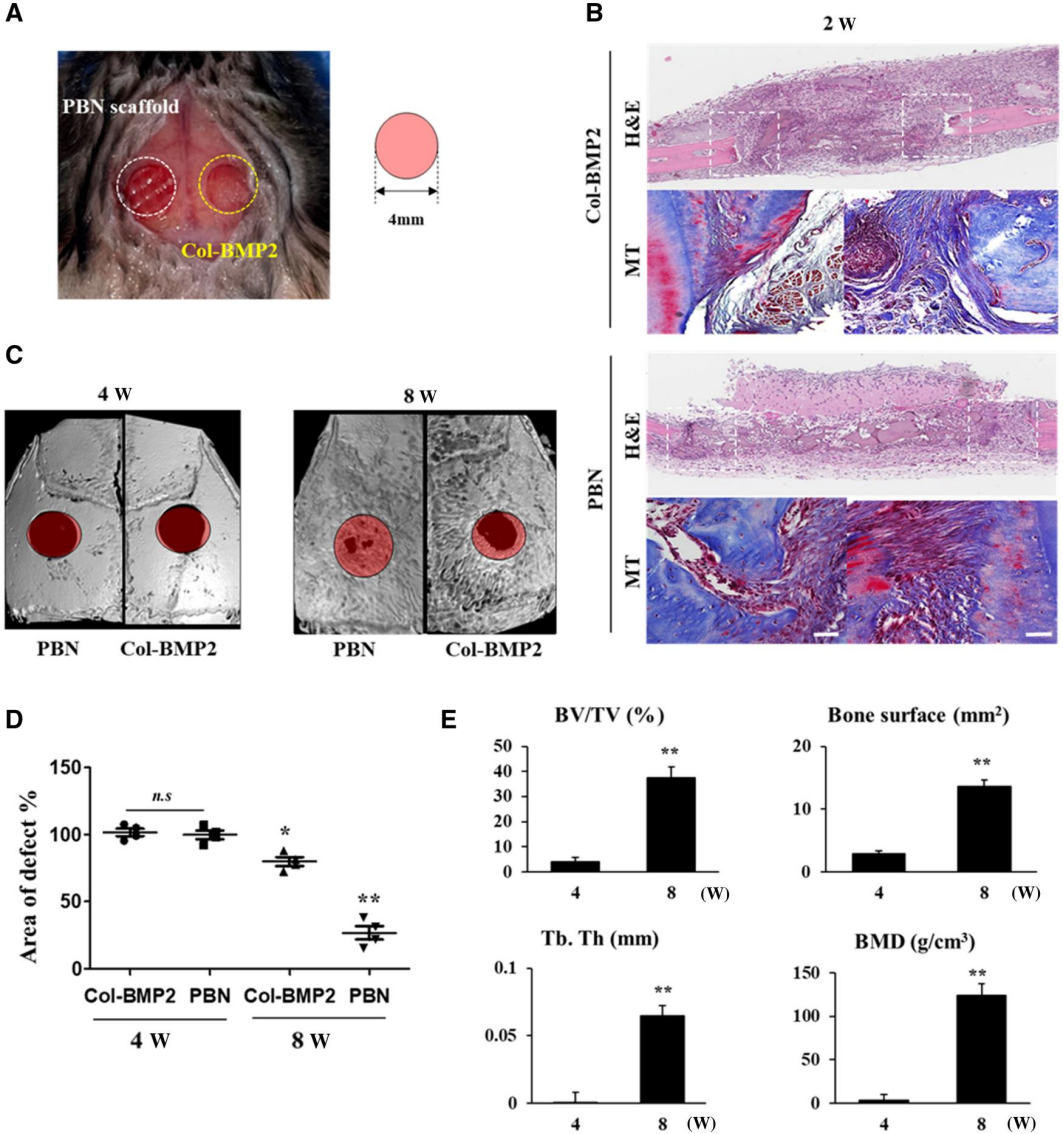
Cavarial bone defect histology and defect regeneration analysis. (**A**) Formation of calvarial defects and experimental group implantation strategy. (**B**) H&E and Masson’s trichrome (MT) staining images 2 weeks after PBN scaffold and control group implantation. In the H&E images, the white box area corresponds to the site of MT staining. Scale = 1 mm. (**C**) MicroCT images 4 and 8 weeks after PBN scaffold and control group implantation. (**D**) Analysis of the defect area ratio shown in microCT. * *P* < 0.05, ** *P* < 0.01, *N* = 4. (**E**) Quantitative analysis of microCT of PBN scaffold implanted group. ** *P* < 0.01. PBN, polycaprolactone-bioink-nanoparticle; H&E, hematoxylin and eosin; microCT, microcomputed tomography.

We verified whether the bone induction ability, confirmed in release modeling and *in vitro* assays, promotes bone regeneration. The regeneration model used a 4 mm critical defect formed in both parietal bones of a mouse, and each defect was applied with a PBN scaffold and collagen sponge absorbed with BMP2 (Col-BMP2) (Figure 6A).

Collagen degrades within 1–2 weeks *in vivo*, causing the initial bust-release of encapsulated drugs and inflammatory reactions. This causes the isogenic bone formation and inflammatory reactions in the soft tissue when collagen sponges are implanted. Numerous inflammatory cells were observed in the Col-BMP2 implant site for up to 2 weeks, and fibrous tissue covering the inflamed area was observed. In contrast, the remaining inflammatory cells in the PBN scaffold were significantly reduced, and the accumulation of extracellular matrix for initial bone formation appeared stronger. In Mason's trichrome staining at the defect interface, fibrous changes were more clearly observed in control than in PBN scaffold (Figure 6B). Microcomputed tomography (CT) scans carried out 4 and 8 weeks after implantation showed no notable difference in bone regeneration between the two groups at the 4-week interval. Nevertheless, a marked difference in bone induction capability became apparent after 8 weeks. By this time, new bone growth was significantly encouraged at the site of PBN scaffold implantation, nearly closing the defect, while only some bone growth was visible at the site of the Col-BMP2 implantation, with the defect remaining unrepaired (Figure 6C). Analysis of the area where the defect had not regenerated into the bone, considering the 4 mm defect as 100%, confirmed that bone regeneration was significantly promoted in the PBN scaffold implantation group (Figure 6D). Quantitative analysis of the micro-CT data from the PBN scaffold group also demonstrated significant bone formation changes from 4 to 8 weeks post-implantation (Figure 6E).

## Discussion

Previous studies have demonstrated strong bone regeneration in animal models using various BMP2 delivery methods, but in humans, a large dose of BMP2 (0.1–1.0 mg BMP2/kg of body weight) is required to induce bone regeneration, which may lead to side effects such as inflammatory or wound complication. Furthermore, difficult conditions commonly found in humans, such as chronic bone nonunion fractures or delayed healing, are challenging to treat with low doses of BMP2, and soft tissue infiltrating the fracture site often impedes bone union [25].

In this study, we utilized a *in vivo* implantable system functionalized with a bone tissue-specific carrier capable of a long-term, stable release of BMP2 using Fibroplex nanocomplexes. We also developed a new scaffold by bioprinting the delivery system into a hydrogel bioink dispersed in a 3D PCL scaffold, which has high biocompatibility while blocking soft tissue infiltration. This continuously releases high doses of BMP2, alleviating suboptimal bone formation and soft tissue inflammation that typically occur due to rapid BMP2 release. We used computer modeling of *in vivo* BMP2 transport to identify the factors that have the most significant impact on BMP2 release, predict scaffold pharmacokinetics that limits BMP2 release into the surrounding soft tissue, and deliver BMP2 maximally to the bone surface. BMP2 release was prolonged in the PBN scaffold, demonstrating the effectiveness of the proposed method. In particular, when the PBN scaffold was implanted at the cranial defect site, robust local bone formation and low ectopic bone formation were observed, with no adverse effects on bone morphology or biomechanics. These results suggest that PBN can improve clinically useful BMP2 delivery methods.

Various ligands have been reported for bone tissue-specific drug delivery; however, the DSS_6_ peptide has ideal bone specificity and chemical binding properties [20, 26]. Considering the drawbacks of bisphosphonate-based ligands, which require non-biocompatible chemical processes and cannot exclude additional osteoclastic effects, the advantages of DSS_6_ are noteworthy. Functionalized nanoparticles using DSS_6_ showed high bone surface-specific retention rates and specificity for hydroxyapatite crystals, among other calcium-based minerals *in vivo*.

Fibroplex, a cationic liposome complex based on fibrin nanoparticles, is a lipoplex family delivery system with various advantages for protein drug delivery [18, 19]. Delivery of BMP2 using Fibroplex has the advantage of maintaining a suitable release rate for bone formation within the defect while reducing the diffusion rate to surrounding tissues and continuously releasing BMP2 at the bone surface. However, many delivery methods, including collagen and alginate, lack control over BMP2 localization on the bone surface *in vivo*. In contrast, the DSS_6_-functionalized Fibroplex demonstrates potent BMP2 retention and release over 4 weeks *in vitro*, does not exhibit rapid initial release effects of the introduced protein, and extends the duration of maintaining physiological concentrations of BMP2.

Bioinks are hydrogel scaffolds formed by the polymerization of biocompatible polymers, primarily used as carriers for bioprinting biomaterials, such as cells and liposomes, which require high biocompatibility. The commonly used gelatin-methyl methacrylate series bioinks undergo a UV-assisted polymerization process, which fails to ensure the stability of Fibroplexes wrapped in liposomes. In this study, the newly developed gelatin-alginate bioink demonstrated a significant increase in the stability of dispersed Fibroplexes owing to its high biocompatibility, CaCl_2_-mediated polymerization and suitable physical properties for bioprinting. Gelatin-alginate bioinks showed high biocompatibility not only with lipoplexes such as Fibroplexes but also when dispersing cells or inorganic materials (hydroxyapatite) and maintained long-term cell survival rates.

PCL scaffolds are thermoplastic polymers with high biocompatibility used for medical purposes for an extended period, such as skull repair. PCL scaffolds can adjust the attachment and survival rates of various cells by controlling the grid and pore sizes. Moreover, the processability and excellent physical properties of PCL make it easy to apply in bone defect areas. In this study, PCL scaffolds were used not only to enhance the applicability of bone-specific functionalized Fibroplexes in bone defect areas but also to optimize the conditions suitable for the attachment and survival of soft tissue by adjusting the grid size of PCL. This prevents the relatively faster-growing soft tissue in the bone defect area from infiltrating the area while also acting as a support to guide the growth of soft tissue.

The PBN scaffolds were simulated to have differential release profiles for soft and hard tissues when implanted at the interface of soft and hard tissues. The release profile of the dispersed particles in the hydrogel bioink was predicted to be concentration-dependent, and the simulation of the dense, soft tissue phase and spatially high permeability of the bone defect area predicted a distinctly higher bone-specific release profile than the collagen sponge. This characteristic was verified through *in vivo* experiments using a skull defect model, which showed an effective induction of bone regeneration within the same period.

## Conclusions

A PBN scaffold that stably supplies physiological concentrations of BMP2 to bone defect areas for an extended period while minimizing the impact on the surrounding soft tissues was developed in this study, and its *in vitro* osteoinductive ability and *in vivo* bone regeneration-promoting ability were confirmed. To deliver and maintain bone-specific BMP2, Fibroplex, a protein drug delivery system functionalized with DSS_6_, was used, and its applicability and stable release were enhanced by dispersing particles in gelatin-alginate hydrogel bioink. The bioink was printed on a PCL scaffold with an improved grid size for soft tissue characteristics forming a characteristic microstructure. These results provide precursor data for PBN scaffolds that can be effectively used for various bone defect diseases such as traumatic bone fractures, osteoporotic bone defects and alveolar bone regeneration due to periodontal diseases.

## Supplementary Material

rbae055_Supplementary_Data
