# Supplementary material for: Bone-targeted lipoplex-loaded three-dimensional bioprinting bilayer scaffold enhanced bone regeneration
Source: Regen Biomater. 2024 Jun 3;11:rbae055. doi: 10.1093/rb/rbae055 (PMC11167398; doi:10.1093/rb/rbae055)

Supplementary Figures

Bone Targeted Lipoplex Loaded Three-Dimensional Bioprinting Bilayer Scaffold Enhanced Bone Regeneration

Supplement Fig. 1 The average size of the DSS_6-Fibroplex

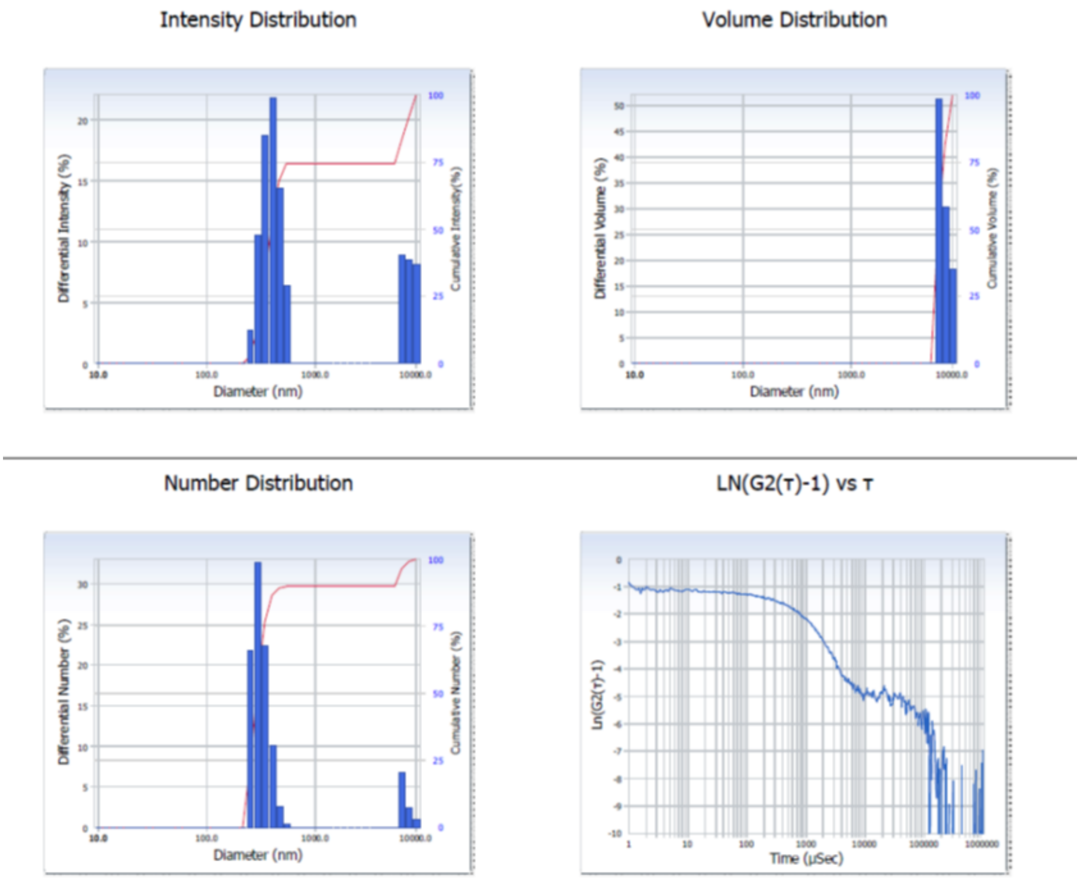

Cumulants Results

Diameter	(d)	: 551.2	(nm)
Polydispersity Index	(P.I.)	: 0.279	
Diffusion Const.	(D)	: 8.925e-009	(cm^2/sec)
Residual		: 1.128e-002	(N.G)

Supplement Fig. 2 *in vitro* loading capacity of rBMP2

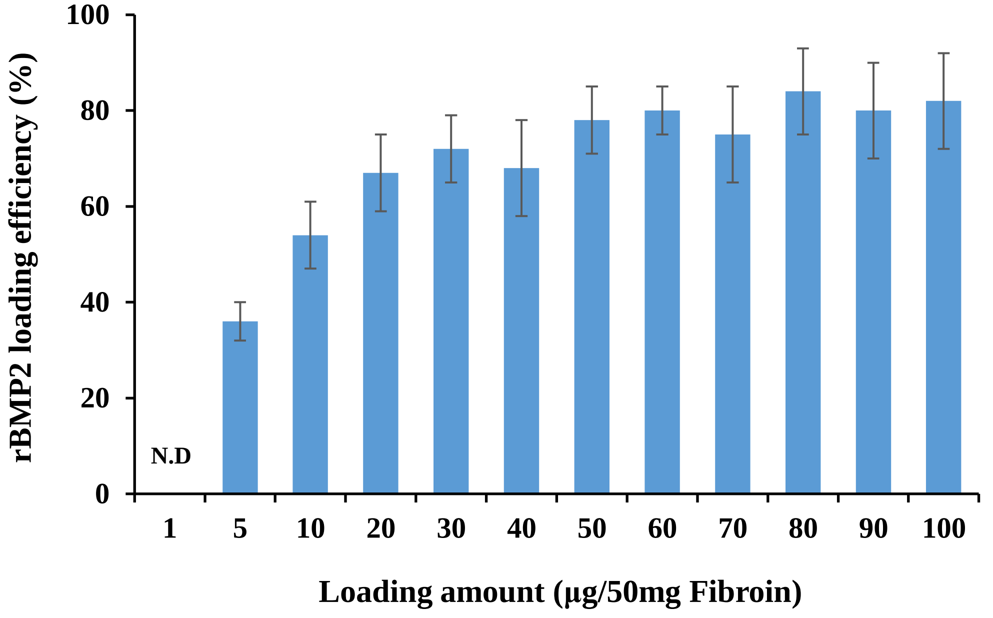

Supplement Fig. 3 Size distribution and Surface potential of rBMP2 loading nanoparticles

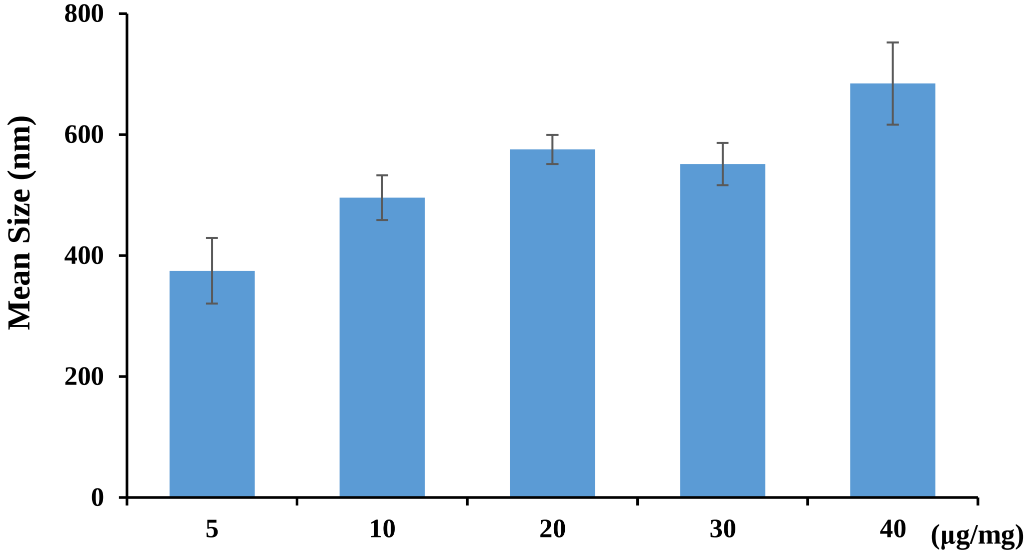

	5	10	20	30	40
**Polydispersity Index (PI)**	0.275	0.331	0.258	0.279	0.301
**Potential**	-37	-35	-38	-31	-42

Supplement Fig. 4 *in vitro* binding capacity of DSS6-modified scaffold to calcium salts

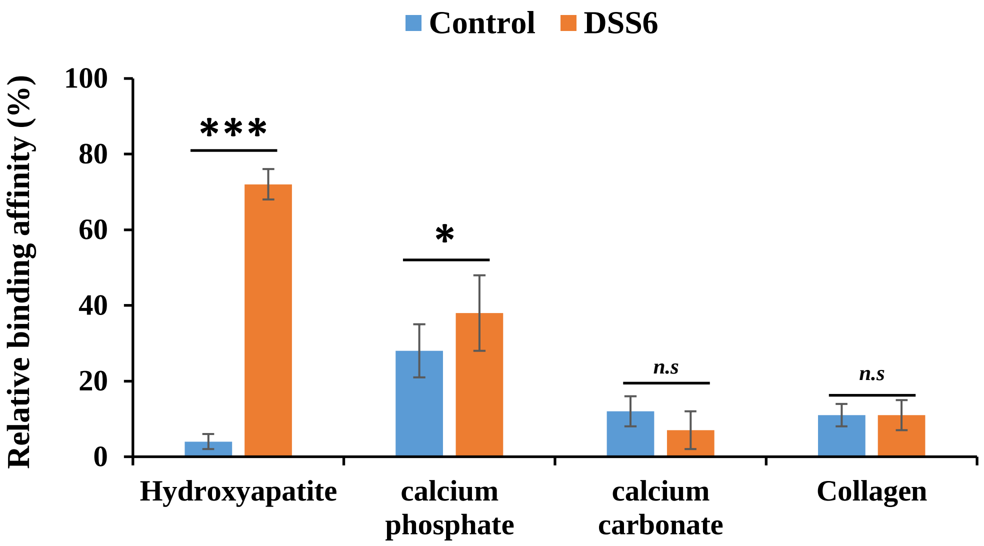

Supplement Fig. 5 viscosity and elastic modulus of DSS_6-Fibroplex laden bioink

a.

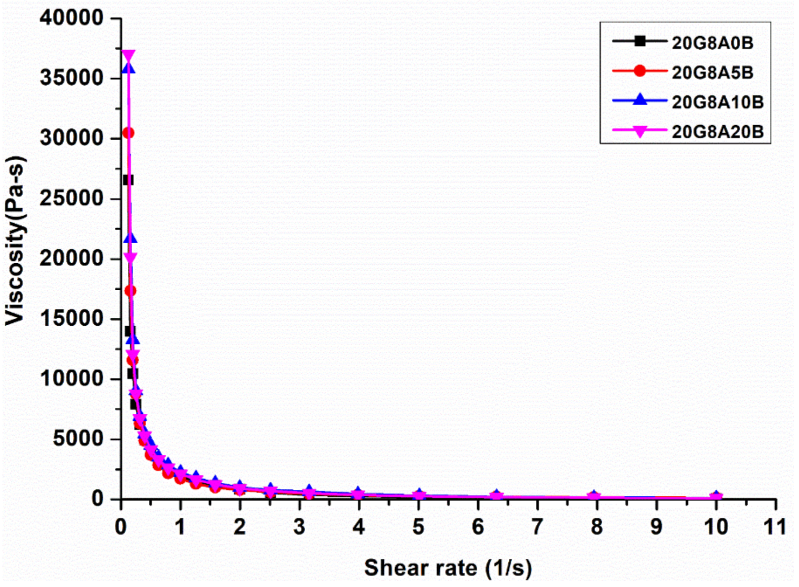

b.

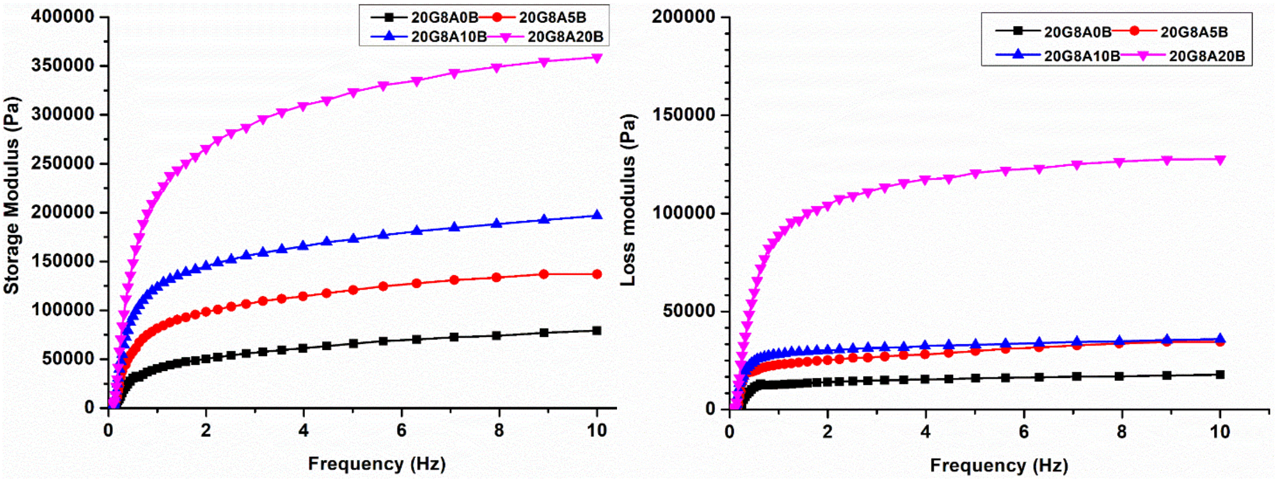

Supplement Fig. 6 Dispersity of DSS_6-Fibroplex laden bioink

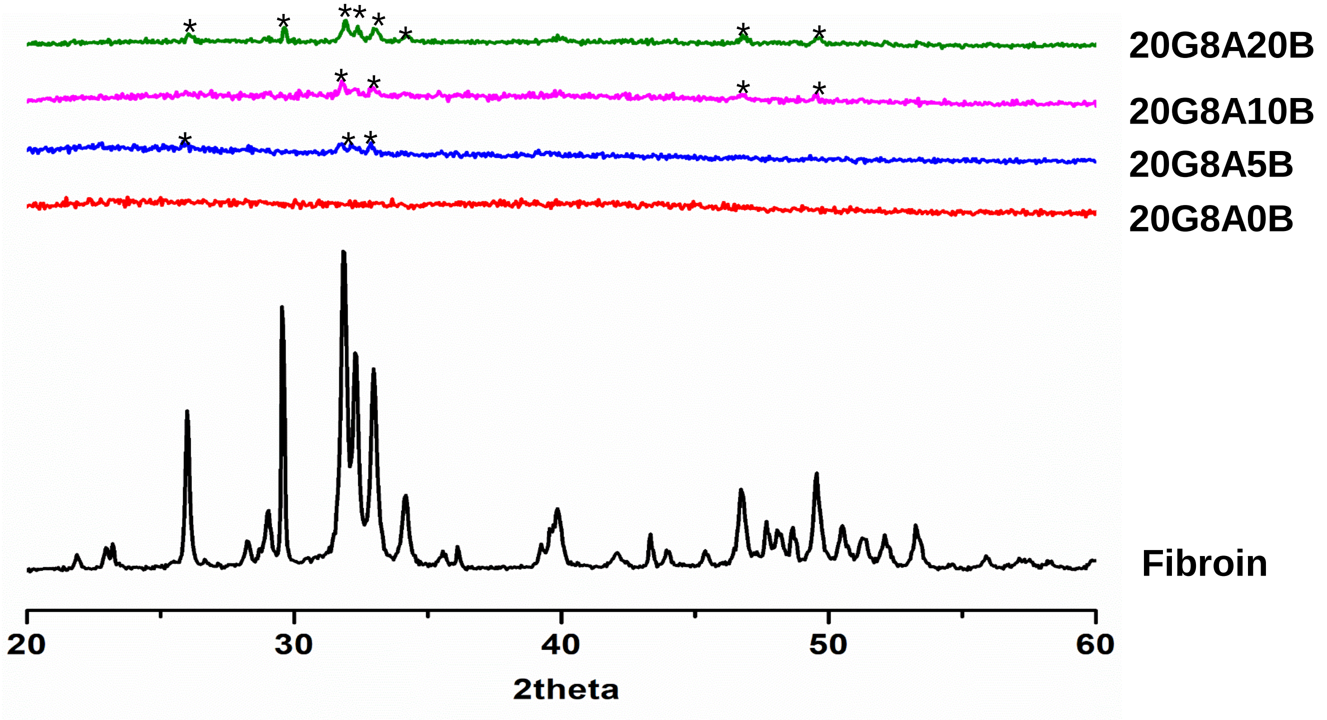

Supplement Fig. 7 Top-view of fluorescence map of bioink

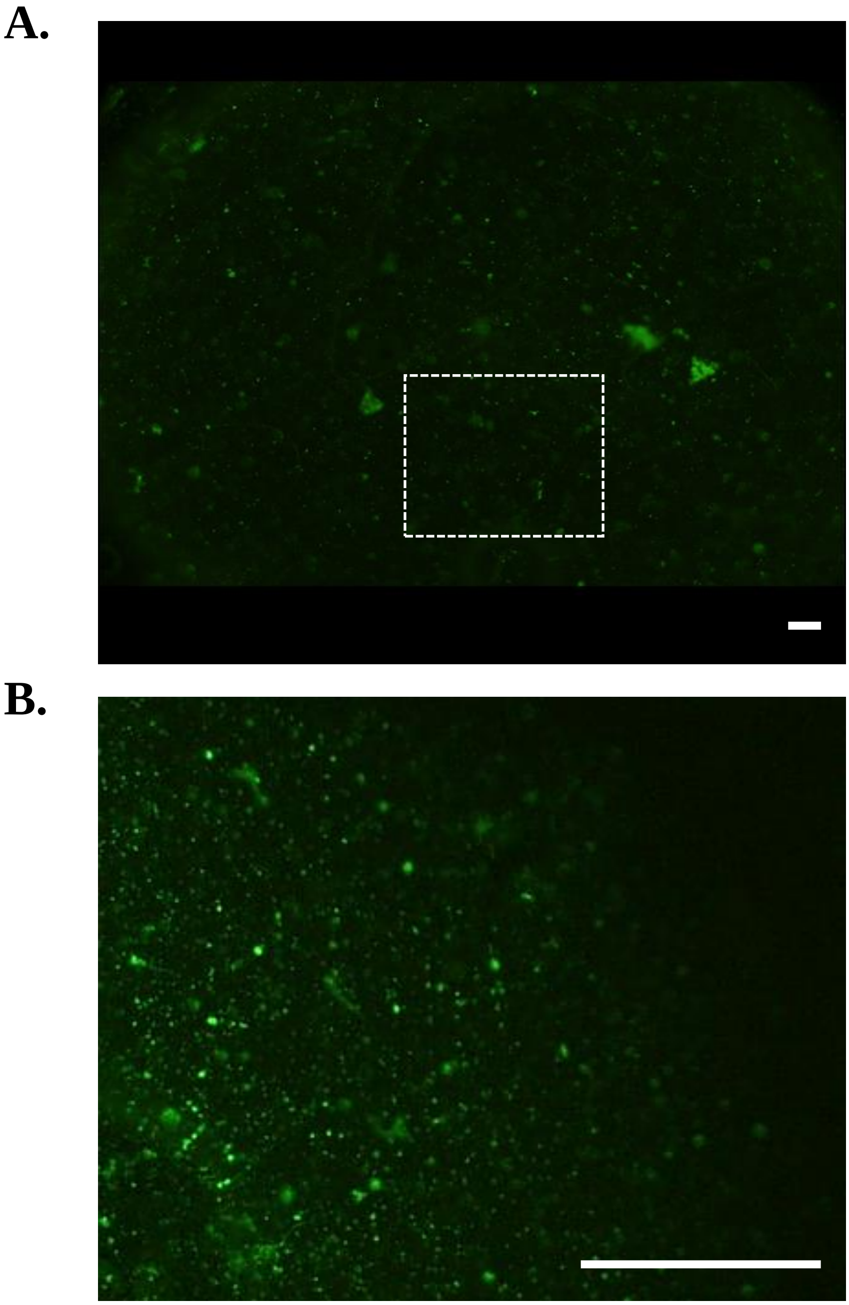

Supplement Fig. 8 Alp staining results of cumulative released BMP2 from PBN scaffold

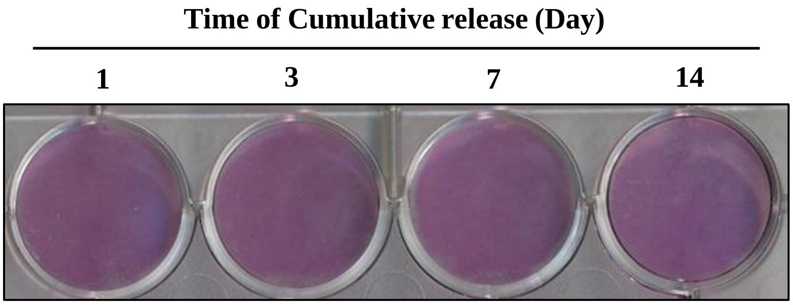

Supplement: rbae055_Supplementary_Data [file rbae055_supplementary_data.pdf]
